# Effect of High Rotational-Speed Friction-Stir Welding on Microstructure and Properties of Welded Joints of 6061-T6 Al Alloy Ultrathin Plate

**DOI:** 10.3390/ma14206012

**Published:** 2021-10-12

**Authors:** Hao Zhang, Shujin Chen, Yuye Zhang, Xinyi Chen, Zhipeng Li, Zhidong Yang

**Affiliations:** School of Materials Science and Engineering, Jiangsu University of Science and Technology, Zhenjiang 212003, China; 192060003@stu.just.edu.cn (H.Z.); 199060077@stu.just.edu.cn (Y.Z.); 201060001@stu.just.edu.cn (X.C.); 192060018@stu.just.edu.cn (Z.L.); yangzhidong@just.edu.cn (Z.Y.)

**Keywords:** high-rotational speed, ultrathin plate, microstructure, mechanical properties

## Abstract

The butt joint of an Al alloy ultrathin plate with a thickness of 0.5 mm is realized by a high rotational-speed friction-stir welding process. It overcomes the welding difficulty that the ultrathin plate is often torn, and it cannot be formed by conventional friction-stir welding. The results show that the weld surface is well-formed at a high-rotational speed (more than 8000 rpm), and there are no obvious defects in each area of the joint section. The nugget zone (NZ) is a recovery recrystallization structure dominated by large-angle grain boundaries, with a grain size of about 4.9 μm. During grain growth, the texture is randomly and uniformly distributed, and the strength is balanced. The microhardness of the NZ increases significantly with the increase in rotational speed, and the fluctuation range of hardness value is small. The NZ β–Mg_2_Si is finer and significantly less than the base metal (BM). The heat dissipation of the thin plate is fast, so a Cu plate is used as the backing plate to slow down the steep temperature-drop process in the weld area. Compared with a low rotational speed, the precipitation amount of brittle phase Al–Cu–Mg–Cr and Al–Fe–Si–Mn is significantly reduced, which is conducive to improving the mechanical properties of the joint. At a high rotational speed, 12,000 rpm, the best tensile strength of the joint is 220 MPa, which is about 76% of the BM (290 MPa), and the highest elongation is 9.3%, which is about 77.5% of the BM (12%). The fracture mode of the joint is a typical plastic fracture.

## 1. Introduction

The demand for intelligence, precision, and miniaturization is increasing in manufacturing industries, most especially in aerospace, microelectronics, and electrical appliances industries. For example, miniaturized precision devices such as aircraft transmitting controllers and signal receiving boxes. Lightweight design is one of the most sought-after solutions and it is widely utilized in manufacturing industries [1,2,3]. One of the promising materials that has emerged is 6061-T6 Al alloy, owing to its combination of excellent corrosion resistance, high strength, high toughness, and good conductivity. These superior properties are responsible for their wide application in the fabrication of aircraft wings and fuselages, automotive rims, and wheel spacers [4,5]. However, it is a huge challenge for improving the manufacturing industry to realize high-quality and efficient aluminum alloy ultrathin plate connections [6,7,8]. Therefore, advanced Laser Beam Welding (LBW) technology and Metal Inert-gas Welding (MIG) were adopted to weld 1 mm 3-Series Al alloy plates. However, the sheet is seriously deformed due to the arc’s high heat-energy input during the fusion welding process, often resulting in insufficient penetration. Moreover, MIG welding requires strict pre-weld cleaning, otherwise there will be pores in the weld, which may affect the joint quality and the complex welding operation [9,10,11]. For an ultrathin Al alloy plate with a thickness less than 1 mm, it is not easy to control the welding heat input and the weld formation is also difficult.

In recent years, friction-stir welding (FSW) has been widely utilized since it has been proven to be a solid-state joining process that is environmentally friendly and cost-effective [12]. The welding process involves no metal melting or crystallization, which avoids welding defects such as blowholes and burning through from the source, and the welding joint remains intact [13,14,15]. FSW is, therefore, regarded as the best choice for thin-plate welding regarding process and cost effectiveness. However, conventional friction-stir welding (CFSW) of sheet metal presents many problems and difficulties, as plastic deformation and metal flow are limited in the weld area of ultrathin sheets [16,17,18]. As thick as the conventional welding tool is, weld flaws such as the formation of keyholes, tunnels, and weak bonding often emerge in forming thin-plate Al alloy joints via CFSW [19,20]. In the process of CFSW of the ultrathin plate, the thick welding tool is easy to produce horizontal tear or serious deformation of the BM, which is due to the thin plate and the limited transverse axial pressure borne by the thick plate welding tool. To obtain a welding joint with small deformation, the welding fixture required for the butt joint of the ultrathin plate is very high [21]. CFSW uses a thick welding tool and large welding load, mainly focusing on the research of plate thicknesses of 2 mm and above, and cannot effectively connect ultrathin plates with weldment thicknesses of less than 1 mm. The thick welding tool and large welding load are major reasons/concerns for poor mechanical properties in ultrathin plates with weldment thicknesses of less than 1 mm.

Several attempts have been made in order to improve the quality of welds produced during FSW of ultrathin plates. A variant of CFSW, high rotational-speed friction-stir welding (HRFSW), sharing similar principles to CFSW, has been developed for its excellent advantages for ultrathin plates’ welding [22,23]. In the actual welding operation, for example, the volume of equipment is doubled, and the geometric structure and size of the welding tool are also different. The welding process window varies with the required plate thickness, which is more in line with the connection requirements of ultrathin plates [24,25]. Relevant research shows that HRFSW technology has been used in the box packaging test of optical fiber transmitters and receivers with good effect. The precision and thinness of the fiber transmitter and receiver boxes indicate that the HRFSW technology has a promising application in the manufacture of thin-plate-type precision instruments. [26,27]. Scialpi et al. [28] and Cerri et al. [29] performed micro-friction-stir welding (μ-FSW) of 0.8 mm-thick sheets of 2024-T3 and 6082-T6 alloys and proved the feasibility and desirability of the μ-FSW in the joining of ultrathin sheets. They elaborated that the μ-FSW joint showed excellent mechanical properties. However, the tensile specimen fractured at the nugget zone (NZ) due to the irregular thickness rather than the existence of defects. Galvao et al. [30] performed μ-FSW of 1 mm-thick sheets of aluminum, copper, copper–zinc, and zinc alloys, and found that the increase in welding speed enhanced process productivity and raised the degree of grain refinement, improving mechanical properties. Panchal, M. et al. [31] successfully connected ultrathin (0.5 mm-thick) commercial Al using μ-FSW technology. They developed a clamping tool suitable for ultrathin sheet processing and changed the rotational speed and welding-speed ratio to obtain ultrathin FSW joints. The results show that the conventional rotational speed and welding-speed ratio cause sheet tearing, and that increasing the rotational speed can improve weld formation. Existing studies have proven the feasibility and desirability of μ-FSW in ultrathin plate connection. However, the spindle speed remains in the low-speed range of 2000 rpm, and the effects of high rotational speed on the formation and properties of the ultrathin sheet have not been studied. At present, the experimental research on 0.5 mm-thick 6-Series Al alloy ultrathin plates using HRFSW technology is scarce. Therefore, research on ultrathin plate connection at high rotational speeds is greatly significant in practical manufacturing.

This paper carried out a butt-joint test of a 0.5 mm-thick 6061-T6 Al alloy ultrathin plate using HRFSW equipment. The effects of high rotational-speed process on the microstructure and properties of the butt joint were examined by optical microscope (OM), scanning electron microscope (SEM), transmission electron microscopy (TEM), and electron backscatter diffraction (EBSD). It is expected to provide a theoretical basis for the application of HRFSW technology in the actual manufacturing of ultrathin light-alloy materials.

## 2. Materials and Methods

### 2.1. Materials

The material used in this study was a 0.5 mm-thick 6061-T6 Al alloy ultrathin plate with the chemical composition shown in Table 1. The plate specification is 150 mm × 80 mm × 0.5 mm.

**Figure 1:**
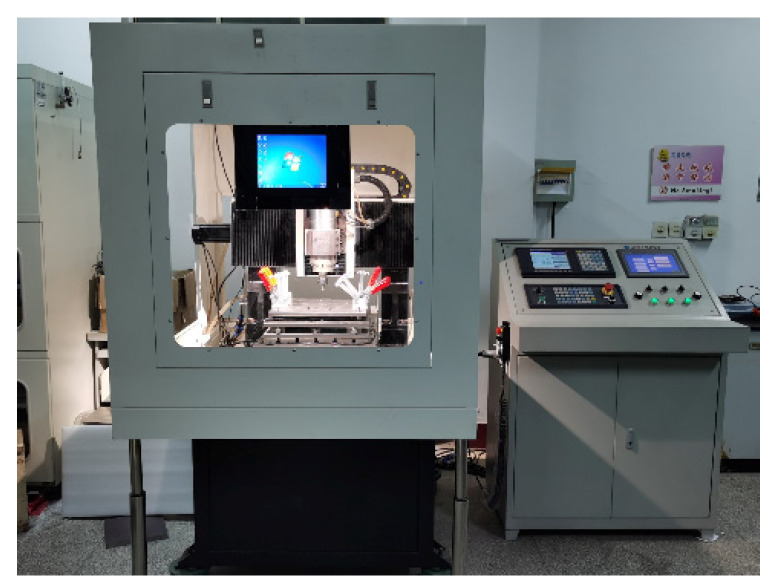
HRFSW machine is required for testing.

**Figure 2:**
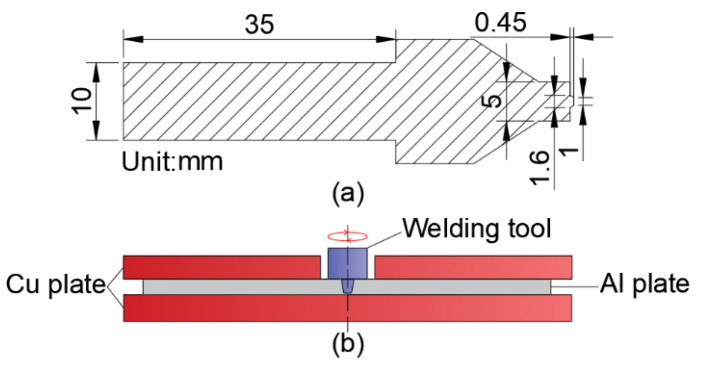
(**a**) Size of the microwelding tool, (**b**) Welding assembly diagram.

The welding machine (The equipment is of our own manufacture) is shown in Figure 1. The maximum speed can reach more than 20,000 rpm. Combined with the characteristics of the HRFSW machine (The equipment is of our own manufacture) and the welding deformation of ultrathin plates, the size design of the welding tool is smaller and more precise. Figure 2a shows the specific size of the microwelding tool (The device is designed by ourselves). The microwelding tool is made of H13 tool steel after heat treatment. H13 tool steel has good strength and hardness, high wear resistance and toughness and excellent comprehensive mechanical properties at high temperatures. It fully conforms to the operation characteristics of friction-stir welding tools (The device is designed by ourselves). Figure 2b shows the welding diagram. The Al plate is mainly placed between two Cu plates for welding. This prevents transverse extrusion deformation of the plate during welding. The Cu plate primarily plays the role of preventing the local temperature from being too high and radiating in time.

### 2.2. Methods

**Figure 3:**
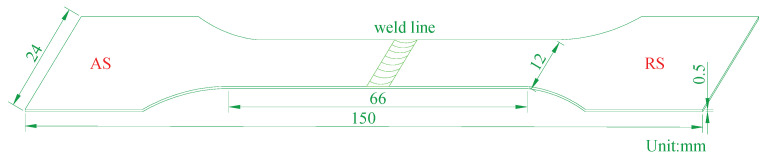
Tensile specimen size.

Before welding, the butt surfaces were ground and cleaned to remove oxides and impurities and to ensure the good formation of the back of the welded joints. The metallographic sample was cross-sectioned perpendicular to the welding direction, electropolished after grinding, then etched in Keller (Xiamen haibiao Technology Co., Ltd., Fujian, China) (95 mL H_2_O + 2.5 mL HNO_3_ + 1.5 mL HCl + 1 mL HF) etchant and observed by microscopy (JSM-6480 scanning electron microscopy, Tokyo, Japan). The microstructure of the NZ was observed by TEM (JEM-2100 transmission electron microscope, Tokyo, Japan) at 200 kV. The samples were prepared by the metallographic method, and then mechanically thinned to 60 μm with 2000 grit sandpaper. Then, the base metal and nugget area are selected to punch into a standard 3 mm diameter wafer, and punching thinning is carried out in a double jet electrolytic thinning device (Leica Microsystems (Shanghai) Trading Co., Shanghai, Ltd., Shanghai, China). The electrolyte is a mixture of 500 mL of 20% perchloric acid and 80% alcohol. The sample is ground to about 1 mm-thick from the end face after wire cutting to remove the cutting damage and oxide layer, then polish with different types of polishing agents until there are no obvious scratches. Finally, SiO_2_ suspension is used for fine polishing to eliminate the surface stress of the sample. The samples were characterized by EBSD system (JSM-6480 scanning electron microscopy, Japan Electronics Co., Ltd., Tokyo, Japan). The EBSD documents were analyzed using Channel 5 software (China electron backscatter diffraction (EBSD) analysis system). The hardness profile was tested on the polished sample across the welded joint using a Vickers hardness tester (KN30S, Suzhou Hengshang Industrial Equipment Co., Ltd., Suzhou, China) with a load of 100 g (HV 0.1) and a dwell time of 20 s. An adequate interval (0.3 mm) was spaced between consecutive indentations to avoid the potential effects of strain fields developed by adjacent indentations. The tensile specimen standard is shown in Figure 3. A WDW microuniversal electronic tensile machine (Jinan metus Testing Technology Co., Ltd., Jinan, China) was used for the tensile test, and the tensile speed was 2 mm/min. The results are taken as the average value of three tests. The welding process parameters are shown in Table 2.

## 3. Result and Discussion

### 3.1. Macro Forming Analysis

**Figure 4:**
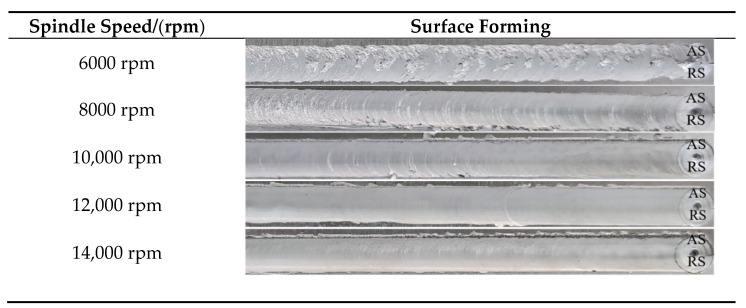
Surface forming of high-speed micro friction-stir weld of the ultrathin plate under different spindle speeds.

Figure 4 shows the weld surface forming under different spindle speeds. When the spindle speed is less than 8000 rpm, the weld surface is rough, and the metal appears in lamellar form on the forward side in a large area. Kissing bond formation is evident on the near surface of the weld. Especially at 6000 rpm, the weld formation is much lower. At a high rotational speed of 12,000 rpm, the weld surface is smooth, the fish scale pattern is clear, the flash on both sides of the weld is reduced, and the weld surface forming is significantly improved. It shows that the welding heat input is insufficient at low speeds. It is difficult to form an extrusion layer between the shaft shoulder and the weld surface, resulting in the low plasticization of the weld ultrathin plate. The weld area does not have sufficient plastic deformation and plastic flow, and it has poor weld surface forming. This explains the phenomenon that ultrathin plates are straightforward to tear at conventional low rotational speeds, as mentioned by Panchal, M. et al. [31]. If the spindle speed increases and appropriate heat input in the weld area is obtained, the extruded plastic metal separates from the BM under the stirring action of the microwelding tool to form a good weld. If the rotational speed is too high, the welding heat input will be too large and material adhesion will increase, which is not conducive to the falling-off of metal materials extruded from the shaft shoulder, resulting in obvious defects on the weld surface.

**Figure 5:**
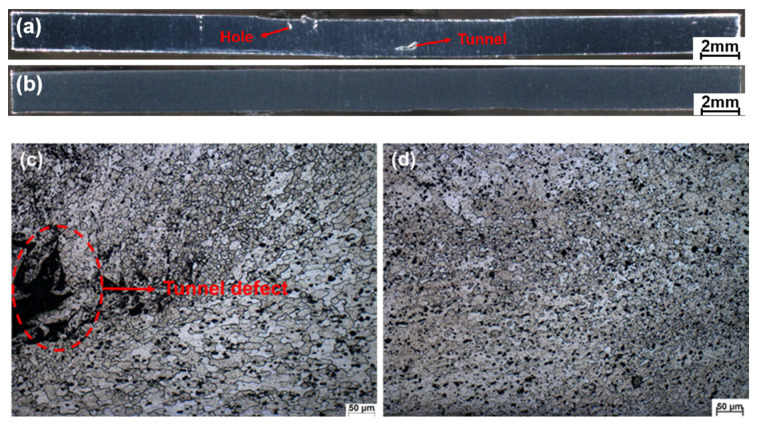
Macro forming of weld section and NZ under different spindle speeds, (**a**) Macro section at 6000 rpm, (**b**) Macro section at 12,000 rpm, (**c**) Micro section of the NZ at 6000 rpm, (**d**) Micro section of the NZ at 12,000 rpm.

Figure 5 shows the macro forming of the weld section and the microstructure of NZ at 6000 rpm and 12,000 rpm. Figure 5a shows that when the spindle speed is 6000 rpm, there are obvious tunnel defects near the bottom of the weld section, hole defects near the surface of the weld, and severe deformation at the bottom of the weld. Figure 5b shows that the weld section is free of defects at a high rotational speed of 12,000 rpm. In order to further observe the defects in the NZ, the microstructure of the NZ was observed. Figure 5c shows that an obvious tunnel is found near the bottom of the NZ, and the tunnel happens to appear at the junction, showing an irregular, layered tearing phenomenon. Figure 5d shows that the structure in the NZ is dense, and that no welding defects are found. The analyses show that under the condition of the low rotational speed, the insufficient welding heat input leads to the insufficient plasticization degree of the weld metal, which is not enough to fill the internal cavity of the weld in the time driven by the mixing head, resulting in keyholes and tunnel defects. The plasticization degree of the weld metal directly affects the magnitude of welding axial force. At low rotational speeds, the weld metal is subjected to low heat input. For the thin plate, the heat dissipation is fast, the plasticization degree is low, and the mixing head has to bear more excellent resistance downward, resulting in severe deformation at the bottom of the weld. Increasing the rotational speed will ensure the weld heat input and promote metal softening in the weld area, and thus form a defect-free and deformation-free welded joint.

### 3.2. Mechanical Property Analysis

**Figure 6:**
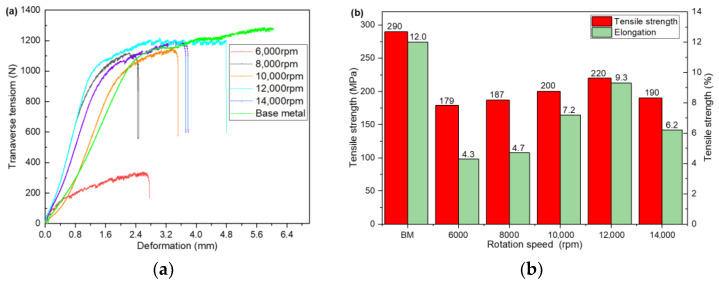
Variation of tensile strength and elongation of joint under different welding parameters, (**a**) The force–elongation curve, (**b**) Variation diagram of tensile strength and elongation.

Figure 6 shows the tensile test results of the joint at different spindle speeds. Figure 6a shows that the tensile deformation of the joint is very different at different rates. Figure 6b indicates that the BM has good plasticity, the elongation after fracture is 12%, and the plasticity decreases after HRFSW. The rotational speed significantly influences the plastic deformation of the joint, and the tensile deformation of the joint is the largest at 12,000 rpm. The tensile strength of the BM is 290 MPa. The elongation of the joint after fracture is consistent with the tensile strength of the joint, showing a trend of “first increase and then decrease” with the increase in the spindle speed. When the spindle speed is 12,000 rpm, the overall mechanical properties of the welded joint are the best. The tensile strength is 220 MPa, which is about 76% of the BM tensile strength. The highest elongation is 9.3%, which is about 77.5% of the BM. The elongation of 77.5% shows that the ultrathin plate weld has good elasticity, which increases the potential application of the FSW joint in the bending and material-forming industry. Under the condition of high rotational speed, the tensile strength of the joint is more than 70% of the BM. A too high or low rotational speed will reduce the tensile strength. At a low rotational speed of 6000 rpm, the elongation of the joint after fracture is only 35.7% of the BM, the tensile deformation is the smallest, and the plasticity becomes worse. Under the condition of low rotational speed, insufficient heat required for the plasticization of the material in the weld area, poor adhesion and welding defects are the main reasons for the obvious reduction in the joint strength, which is consistent with the results described in Figure 5. On the other hand, the joint strength may be related to the change of precipitates in the weld area, which needs further analysis. If the rotational speed is too high and the heat input is too high, the microstructure in the nugget area is coarsened and the performance of the joint is reduced. At the appropriate rotational speed, the tensile strength and joint plasticity will be significantly improved.

**Figure 7:**
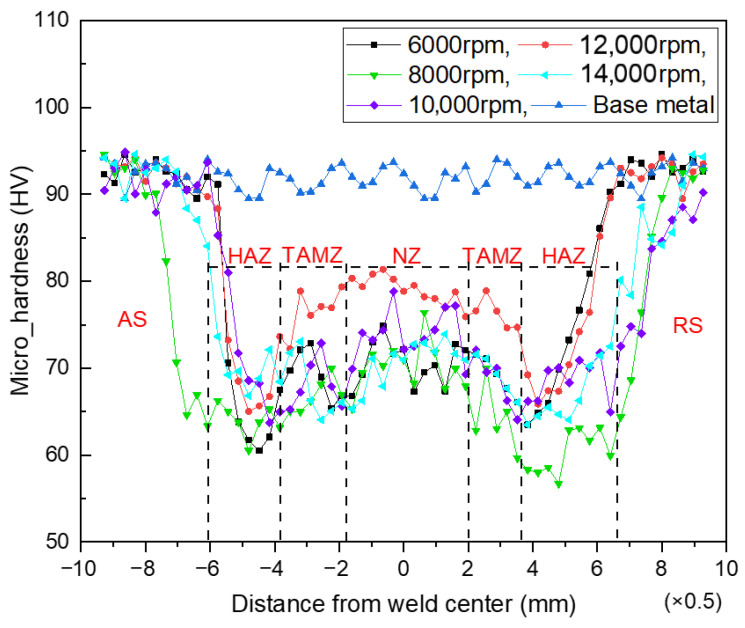
Variation curve of microhardness at different rotating speeds.

Figure 7 shows the hardness variation curve of the joint area under different spindle speeds. The measured average hardness of the BM is 92 HV. Figure 7 indicates that the width of the NZ is about 4 mm, and the shape of the microhardness curve in the joint area is W-shaped. The results show that each joint region softens in different degrees under the influence of the microwelding tool, which makes the joint undergo the dual effects of mechanical stirring and extrusion. The changing trend of microhardness in each joint region is different, and the microhardness in the weld zone will show a downward trend compared with the BM. The maximum microhardness of the NZ is 74 HV, which is only 80.43% of the BM. With an increase in rotational speed, the overall hardness of the weld zone of the joint increases, and the hardness value of the NZ tends to be flat.

When the rotational speed is 12,000 rpm, the microhardness of the NZ of the joint is higher. The maximum microhardness is 81.35 HV, which is 88.42% of the average microhardness of the BM. Compared to with the low rotating speed, the hardness is increased by 8%. The microhardness of the heat-affected zone (HAZ) on the forward side (AS) is the lowest. The analysis shows that the metal plasticization degree in the weld area is low at low rotational speeds, and there are welding defects such as holes and tunnels, which cannot form a good joint. The microhardness fluctuates wildly, and the overall hardness value is small, which is consistent with that described in Figure 5a. When the rotational speed is appropriate, the welding heat input is sufficient, the plastic metal flows fully, and the weld formation is good. The microstructure of the NZ is uniform and refined, the joint strength is naturally improved, and the fluctuation range of hardness is gentle. The hardness change is not only affected by the weld structure but is also related to the shift of precipitates in the weld area. In 6061-T6 Al alloy, precipitation hardening in the plate plays an important role in analyzing microhardness height. Under the condition of high rotational speed, the microhardness of the NZ is slightly lower than that of the BM, which may be due to the dissolution of more precipitates in the matrix.

### 3.3. Precipitation Phase Analysis

**Figure 8:**
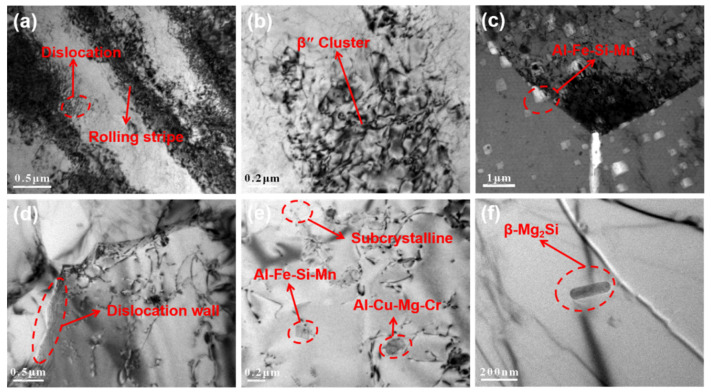
Transmission electron microscope of the BM and NZ, (**a**,**b**) BM, (**c**) NZ at 6000 rpm, (**d**–**f**) NZ at 12,000 rpm.

**Figure 9:**
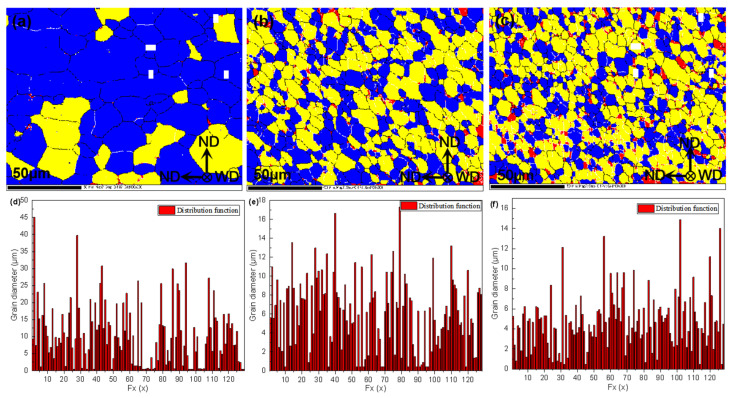
Microstructure, morphology and grain size, (**a**,**d**) BM, (**b**,**e**) NZ at 6000 rpm, (**c**,**f**) NZ at 12,000 rpm.

Through the above analyses, it was found that the welding defects are the main reasons for the change of joint strength. The joint strength also is closely related to the number, distribution, and composition of precipitates throughout the above analysis. The existence of subcrystal structure and dislocation in the weld zone may account for the decrease of joint elongation. Figure 8 is a TEM photograph of the BM and NZ of the joint under the conditions of low rotational speed, 6000 rpm, and high rotational speed, 12,000 rpm. In Figure 8a, obvious rolling stripes of 6061-T6 can be seen from the microscopic point of view. During the rolling process, the grains are deformed strongly. Different degrees of dislocation walls can be seen along the grain boundary, which is mainly caused by the concentration of plastic resistance of the metal during the plastic deformation of the BM during the rolling process. Figure 8b shows the cluster phenomenon of the precipitation strengthening phase in the rolling stripe, formed by the enrichment of large particles (Mg, Si) of 6061-T6 Al alloy in the rolling state along the rolling direction. The enriched (Mg, Si) large particles are mainly acicular β″. The phase form exists, and the particle size and quantity are relatively large, which is consistent with the results of Du, C. [32]. There are a lot of rolling strips in the microstructure of 6061-T6 Al alloy, which is enriched with a certain density along with the rolling direction β″ Phase, and these β″ Phases in the BM play an important role in strengthening the matrix, which makes the BM show excellent mechanical properties [33]. Mg_5_Si_6_ (β″) is the most effective metastable hardening precipitate in Al–Mg–Si (6-Series) alloy. It belongs to the metastable GP2 zones in the ductile matrix, which can hinder the dislocation motion and thus strengthen the materials by increasing the hardness [34,35]. Figure 8c shows that many square insoluble brittle Al–Fe–Si–Mn phases appear in the NZ at 6000 rpm. The brittle phase is distributed not only in the crystal but also along the grain boundary. No evident dislocation phenomenon was found at the grain boundary, which shows that the NZ is mainly mechanically stirred by a microstirring tool and is less affected by shear force at low rotational speeds. The generation of these brittle phases is also one of the reasons for the further reduction in the strength of the joint. Figure 8d shows that high-density dislocation walls appear along the grain boundary in the nugget region at 12,000 rpm. This is because the ultrathin plate is broken and renucleated after high-speed stirring by the microwelding tool. At the same time, it is deformed under the action of shear force. The dislocations in the deformation process are highly accumulated to form a dislocation wall and are attached to the grain boundary. Figure 8e shows that the intersection of subgrain structure and dislocation occurs at the grain boundary, which is mainly due to the nonequilibrium subcrystal boundary formed by dynamic recrystallization and dislocation absorption in the nugget area under the action of thermal coupling [36]. The analysis shows that the subcrystal structure in the NZ and the existence of high-density dislocations may be the reason for the decrease in joint elongation compared with the BM. Figure 8e and Figure 9f show that there are a large number of needle-shaped particles in the grain boundary of the NZ β″ Phase, and rod-shaped β–Mg_2_Si, distributed and dispersed. Compared with low speeds, the square insoluble Al–Fe–Si–Mn phase is significantly reduced, and a small amount of elliptical brittle Al–Cu–Mg–Cr phase appears. The analysis shows that the increase in shear force in the NZ during high-speed welding is affected by the high-temperature thermal cycle and severe plastic deformation. This causes major β-Phase transition and remelting, plus the strengthening phase redistributes, and the degree of uniformity is much less than the BM. After ultrathin plate welding, the heat dissipation inside the weld is fast, forming a specific temperature gradient. The thermal conductivity of the Cu plate makes the temperature change more slowly. During the cooling process of the NZ, the precipitation amount of brittle Al–Cu–Mg–Cr phase and square Al–Fe–Si–Mn phase decreases. Therefore, under the condition of high speed, the strength of the joint is higher than that at low speed. On the one hand, the welded joint has no defects under the condition of high speed; on the other hand, the number of brittle phases is less than that at low speed. However, the generation of brittle phase is an unfavorable factor for the change of mechanical properties of the joint.

### 3.4. Microstructural Analysis

Figure 9a shows the essential characteristics of the BM. Most grains are composed of coarse and flat deformed structures when viewed perpendicular to the rolling direction. In the rolling state, the plastic deformation of the BM structure is more serious, leading to the formation of a large number of dislocations. Some dislocations gather and recombine during plastic deformation to form dislocation walls. The dislocation walls continue to absorb dislocations and gradually transform into small-angle grain boundaries. Figure 9c shows that the average grain diameter of the BM is 10.47 μm. Figure 9b shows the morphological characteristics of the NZ after stirring with the microwelding tool at 6000 rpm. It can be seen that the grains in the NZ are composed of refined equiaxed grains with uniform grain size, and there are no noticeable coarse grains [37,38]. Compared with the microstructure characteristics of the BM, it can be found that the morphological structure of the NZ decreases while the proportion of subcrystalline structures and recrystallized grains increases significantly. Figure 9e shows that the average grain size in the NZ is 6.54 μm. Figure 9c shows the morphological characteristics of the NZ after stirring with the microwelding tool at 12,000 rpm, and compares it with the microstructure characteristics of the BM. The results show that the grains in the NZ are more refined and more evenly distributed. The morphologic structure of the NZ was further reduced, while the proportion of subcrystalline structures and recrystallized grains increased. Figure 9f shows that the average grain size in the NZ is 4.94 μm. This is mainly because the grains in the NZ bear the rotating friction and shear force of the microwelding tool during high rotational-speed welding. The plastic metal has a relative movement along the welding direction under shear force, and its flow speed and direction change with high rotational-speed welding. Due to the velocity gradient of the relative movement of the plastic metal under the shear force, the plastic material around the stirring tool changes dynamically and randomly during the stirring process. In this process, the dislocation density increases continuously, and the stored energy at the grain boundary accumulates constantly. When the stored energy rises to the point where recrystallization can occur, crystal nuclei are generated inside the metal, and the crystal nuclei are mechanically broken before they grow up. The aggregation phenomenon of dynamic recovery and recrystallization of grains and substructure grains is formed at the grain boundary, transforming it from a small-angle grain boundary to a large-angle grain boundary [39,40]. This is consistent with the description in Figure 8e. It also proves that the microstructure formation mechanism of the NZ of 6061-T6 Al alloy ultrathin plate HRFSW joint is continuous dynamic recrystallization. Grain size has an important influence on its mechanical properties. Figure 7 shows that the hardness of the NZ is higher than that of the HAZ and the TMAZ. The main reason is that under the condition of high rotational speed, the grains in the NZ have significantly changed compared with those in the BM, the grain orientation is uniform, and the grains are refined and dense. The NZ plays the role of fine-grain strengthening and maintains the mechanical properties of the welded joint.

**Figure 10:**
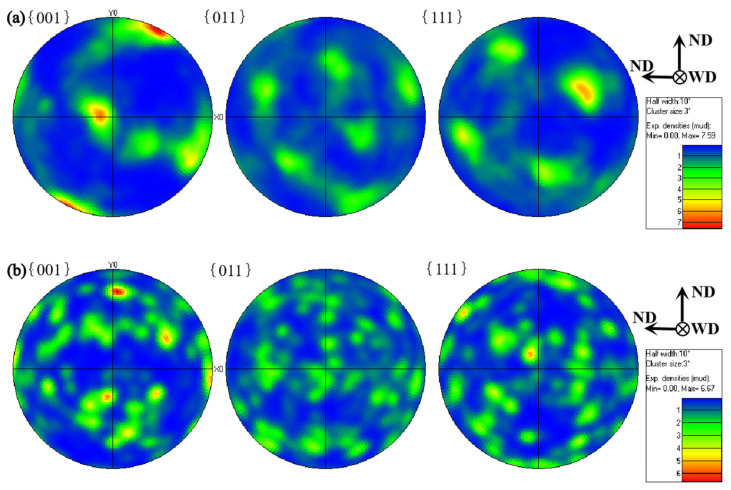
Pole diagram, (**a**) BM, (**b**) NZ at 12,000 rpm.

Figure 10 shows the texture changes of ultrathin aluminum alloy in the BM and NZ grain during growth under the condition of HRFSW. Figure 10a shows that for 6061-T6 rolled Al alloy, {001} the crystal plane is always parallel to the WD crystal plane, and the texture of the main plane of the BM is {001} < 100 >, with remarkable texture characteristics. Relevant studies show that in the welding zone of 6061 Al alloy after conventional FSW, the texture usually becomes a plane texture of {111} < 110 > and {001} < 110 >, and the texture growth has a certain directionality [32,33,34,35]. On the contrary, the texture in the NZ of ultrathin Al alloy plates induced by HRFSW grows randomly, the texture strength is balanced, and the orientation dispersion is very small. Figure 10b shows that the crystal-plane texture distribution of NZ is uniform and parallel to the WD direction. It is mainly a little {112} < 110 > B texture. This fully shows that the grain stirring in the NZ after HRFSW is sufficient. It explains the formation of finer and uniform equiaxed grains in the NZ at high speed. The strengthening effects of fine grain in the NZ are obvious, and the fluctuation range of microhardness is minimal.

### 3.5. Tensile Fracture Analysis

**Figure 11:**
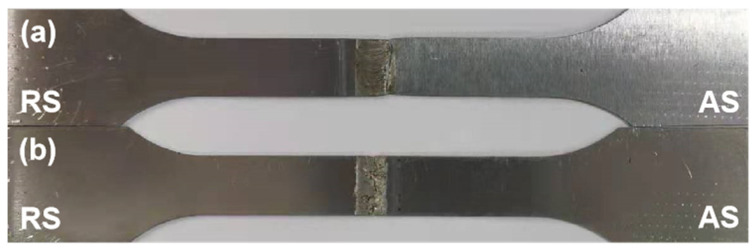
Tensile fracture, (**a**) breaking position at 12,000 rpm; (**b**) breaking position at 6000 rpm.

Table 3 shows the different fracture forms of the joint at different speeds. Figure 4 shows that when the rotational speed is 6000 rpm and 8000 rpm, the heat input is small, and the material fluidity is poor. Under the action of the microwelding tool, the weld has a downward movement speed component, and there is a difference between the horizontal rotation speed and the low movement speed. The stirring pin cannot timely drive the plasticized metal to fill the cavity on the retreating side, so it cannot be formed, and it is prone to welding defects such as holes, tunnels, and obvious weak bonding defects on the near surface of the weld. This directly causes the joint to break in the weld center, as shown in Figure 11a. When the rotational speed is increased to make up for the insufficient welding heat input, the plasticized metal flows more fully, the defects disappear, and the joint strength is improved. However, the metal accumulation layer is appears easily at the junction of the HAZ and TMAZ. The grain size is larger than the NZ, so it is easy to fracture on the forward side. When the rotational speed is too high, the excessive welding heat input results in reduced viscous friction of the joint. High rotational speed of the mixing causes slippage with the weld metal. As shown in Figure 11b, the thickness of the thin plate makes the weld metal unable to be effectively combined and makes it easy to fracture in the weld center.

**Figure 12:**
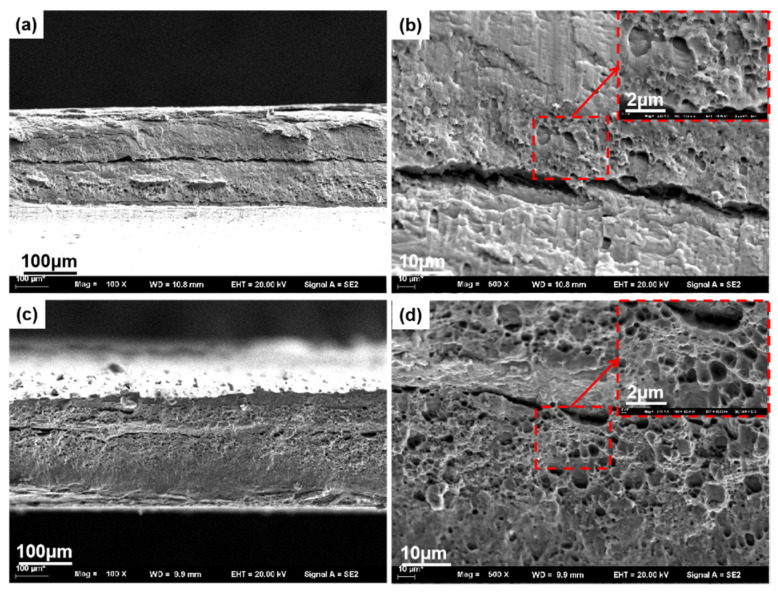
Fracture morphology of samples under different magnification, (**a**,**b**) Fracture morphology under different multiples at 6000 rpm, (**c**,**d**) Fracture morphology under different multiples at 12,000 rpm.

Figure 12 shows the fracture micromorphology of the two groups of tensile specimens with the lowest and highest elongation. Figure 12a,b shows few dimples in the fracture morphology under low elongation, which are mainly distributed in the center of the weld section. More lamellar tearing edges are observed, the fracture is flat, and the delamination phenomenon is evident in the center of the area [1,41,42,43,44]. It shows a kissing bond in the joint at low rotational speed and a typical brittle fracture, which coincides with the results in Figure 12a. The mechanical properties of the joint are the lowest, which is consistent with the results in Figure 6a. Figure 12c,d shows a large number of dimples of uniform size, and the large dimples also contain many small dimples. The depth of the dimples are shallow, and the torn edge tends to be flat and smooth. The second phase particles at the bottom of the dimple are also more refined. The fracture is a typical plastic fracture, which is in keeping with the results of postfracture elongation and has good plasticity. By comparing the fracture surfaces of samples with different elongations, the fracture dimples with high elongation are more evenly distributed and higher in number. The fracture with low elongation is a brittle fracture with low strength. Therefore, the tensile and fracture mechanical properties at high rotational speeds are better.

## 4. Conclusions

In this study, a microwelding tool suitable for ultrathin plate welding is designed. On this basis, the butt-joint test of a 6061-T6 Al alloy ultrathin plate is carried out using HRFSW. The changes in microstructure and properties of ultrathin plate joints under HRFSW were analyzed. The main conclusions are as follows:

1. Low rotational-speed welding of a 0.5 mm-thick Al alloy ultrathin plate will have welding defects such as holes, tunnels, and weak bonding. HRFSW can obtain high-quality welded joints. When the spindle speed is 12,000 rpm and the welding speed is 300 mm/min, a joint with a good weld surface and no obvious welding defects is obtained.
2. The mechanical properties of the welded joints obtained by HRFSW are significantly improved compared with those obtained at low rotational speeds. The tensile strength is about 76% of the BM. The average microhardness of the NZ is approximately 88.42% of the BM. The plastic deformation of the joint is the largest, the joint has good plasticity, and the elongation after fracture is about 77.5% of the BM.
3. Under a high rotational-speed process, the grain size of the joint welding area decreases significantly after mechanical stirring with a microwelding tool. The microstructure is mainly composed of fine recovery recrystallization grains and subgrain structures, which plays the role of fine-grain weld strengthening. The grain texture grows randomly during the growth process, and the texture strength is relatively uniform, indicating that the high rotational-speed microwelding tool can stir more fully and evenly in the NZ. Due to the thin plate thickness, fast heat dissipation, and large temperature gradient in the NZ, there is a small amount of insoluble brittle phase in the weld area in addition to the dispersed Mg_2_Si. The precipitation of the brittle phase is the main factor causing the joint strength to be lower than the BM.
4. The welded joint obtained by HRFSW has large deformation and high elongation. The dimples on the fracture surface are dispersed and higher in number, which are typical of plastic fractures with better mechanical properties; at low rotational speed, the elongation and strength of the joint are low, and the fracture is brittle.

## Figures and Tables

**Table 1:** Chemical composition (wt%) of 6061-T6 Al alloy.

Si	Fe	Cu	Mn	Mg	Cr	Zn	Ti	Al
0.40–0.8	0.7	0.15–0.04	0.15	0.8–1.2	0.04–0.35	0.25	0.15	allowance

**Table 2:** Welding process parameters.

Factors	Spindle Speed (rpm)	Welding Speed (mm/min)	Plunge Depth (mm)
1	6000	300	0.03
2	8000	300	0.03
3	10,000	300	0.03
4	12,000	300	0.03
5	14,000	300	0.03

**Table 3:** Fracture position of joint at different speeds.

Serial Number	Spindle Speed (rpm)	Elongation (%)	Fracture Location
1	6000	4.3	Weld center
2	8000	4.7	Weld center
3	10,000	7.2	RS
4	12,000	9.3	RS
5	14,000	6.2	Weld center

## Data Availability

Data sharing not applicable. No new data were created or analyzed in this study. Data sharing is not applicable to this article.
